# Drug Reaction With Eosinophilia and Systemic Symptoms (DRESS) Due to Trimethoprim: A Case Report

**DOI:** 10.7759/cureus.94282

**Published:** 2025-10-10

**Authors:** Hammad Buksh Ilahi, Maheen Bhangwar Baloch, Eirini Kasfiki

**Affiliations:** 1 Medicine and Surgery, Ziauddin University, Karachi, PAK; 2 Internal Medicine, Hull University Teaching Hospitals NHS Trust, Hull, GBR

**Keywords:** cutaneous adverse drug reaction, drug-induced hypersensitivity, drug-induced pneumonitis, drug reaction with eosinophilia and systemic symptoms (dress), pulmonary complications, trimethoprim

## Abstract

Drug reaction with eosinophilia and systemic symptoms (DRESS) is a rare, severe, and potentially life-threatening drug hypersensitivity reaction characterized by cutaneous eruption, hematologic abnormalities, and internal organ involvement. Early recognition and prompt withdrawal of the culprit drug are crucial to reduce morbidity and mortality. We report a case of a 17-year-old woman with a psychiatric history who presented with a progressive maculopapular rash, fever, cough, and lethargy after initiation of trimethoprim for acne. Examination revealed a diffuse rash and bilateral chest crepitations. Laboratory studies showed elevated inflammatory markers, eosinophilia, and deranged liver function tests. Imaging demonstrated bilateral pulmonary infiltrates. Initial improvement was noticed with oral steroids, but her symptoms recurred on withdrawal. After which, corticosteroids were introduced, but despite this, her respiratory function deteriorated, necessitating intensive care admission. Microbiological investigations were negative for infection. Based on clinical features and drug association, along with European Registry of Severe Cutaneous Adverse Reaction Criteria (RegiSCAR) scoring, a diagnosis of DRESS syndrome secondary to trimethoprim, further complicated by pneumonitis, was made. The patient was treated with systemic corticosteroids with gradual clinical and biochemical improvement over six weeks. This case highlights trimethoprim as a potential but under-recognized trigger for DRESS syndrome. Early identification, discontinuation of the offending drug, and careful use of corticosteroids are essential to prevent severe complications. Clinicians should maintain a high index of suspicion for DRESS in patients presenting with rash, fever, eosinophilia, and multiorgan involvement following recent drug exposure.

## Introduction

Drug reaction with eosinophilia and systemic symptoms (DRESS) is a rare and severe hypersensitivity reaction that mainly involves the skin, but can have widespread multisystem organ involvement [1]. The disease is a life-threatening condition with cutaneous presentation and internal organ involvement, and the mortality rate being 10% [2]. The timeline for the development of symptoms varies from two to six weeks after initiation of the culprit drug. The initial symptoms are usually fever and a maculopapular rash that may progress to exfoliative dermatitis. Hepatitis, lymphadenopathy, hematologic abnormalities, and renal dysfunction are observed with varying degrees and are associated with significant morbidity and mortality [3,4]. The diagnosis is difficult as the multiorgan involvement causes a broad clinical spectrum [4]. It is essential to recognize it promptly and discontinue the culprit drug. The European Registry of Severe Cutaneous Adverse Reaction Criteria (RegiSCAR) was developed in 2007, which developed inclusion criteria as well as a scoring system classifying suspected cases as no, possible, probable, or definite DRESS case [5]. Drugs at high risk of causing DRESS are especially aromatic anticonvulsants (e.g., carbamazepine, phenytoin, lamotrigine), allopurinol, and antibiotics, such as beta-lactams and vancomycin [5]. The latency period between initiation of medication and appearance of symptoms varies between one and eight weeks.

## Case presentation

A 17-year-old woman with a background history of psychiatric illness, including previous paracetamol overdose and eating disorders, presented to the Emergency Department with a maculopapular rash, shortness of breath, and generalized malaise. The rash initially appeared on her arms and legs before spreading to the chest and trunk. It was pruritic in the early stages. She also reported non-productive cough, reduced appetite, and increasing lethargy.

Her past medical history was significant for acne, for which she had been on doxycycline from January to March. During a holiday in Italy in March, her acne worsened, and she was subsequently commenced on trimethoprim. Shortly after initiation, she developed the rash, which followed the described progression. She was reviewed by her general practitioner, who prescribed oral steroids, leading to temporary improvement. However, following cessation of steroids, the rash recurred and worsened.

She had no previous history of drug allergies, no recent changes in diet, and denied any alcohol or recreational drug use. There was no significant family history, including no known atopy or allergic disorders. She had recently traveled to Switzerland and France but had no history of animal contact. We applied the RegiSCAR validation scoring system (see Table 1) to classify cases--scoring ≥6 confirmed definitive DRESS, while 4-5 indicated probable DRESS [6].

**Table 1:** RegiScar scoring criteria. DIHS: Drug-induced hypersensitivity syndrome, DRESS: drug reaction with eosinophilia and systemic symptoms; RegiSCAR: European Registry of Severe Cutaneous Adverse Reaction Criteria. Adapted from Ref. [6].

Parameter considered by Japanese consensus group criteria	The possible manifestations (of each parameter) that can satisfy diagnosis of DIHS	Point on RegiSCAR DRESS validation scoring system for features of atypical/typical DIHS	Minimum and maximum points that can be obtained by a patient with atypical/typical DIHS on RegiSCAR/DRESS validation scoring system (minimum)	Minimum and maximum points that can be obtained by a patient with atypical/typical DIHS on RegiSCAR/DRESS validation scoring system (maximum)
Latent period between onset of drug intake and appearance of symptoms	More than three weeks	0	0	0
Duration of clinical symptoms after withdrawal of the offending drug	Prolonged clinical symptoms after withdrawal of the offending drug	0	0	0
Fever	≥38°C	0	0	0
Maculopapular rash	1. Maculopapular rash involving >50% of body surface area and suggesting DIHS features on skin biopsy; 2. Maculopapular rash and infiltration of the features suggesting DIHS (facial edema, purpura on limbs excluding sun-exposed areas, scaling, and purpuric lesions)	1 point for generalized maculopapular rash and -1 point for rash not showing, ≥2 features suggestive of DRESS. Net score 0. +1 point for generalized maculopapular rash and +1 point for rash showing, ≥2/4 features suggestive of DRESS. Net score 2	0	2
Internal organ involvement	1. Liver or other organ involvement. 2. Two or more internal organ involvement	0–2	0	2
Hematological criteria	1. Leukocytosis ≥11,000 cells/mm³; 2. Atypical lymphocytes in peripheral smear; 3. Absolute eosinophil count >1500 cells/mm³	Leukocytosis +0; Atypical lymphocytes +1; Eosinophilia +1 or +2	0	3
Cervical/generalized lymphadenopathy	1. Cervical lymphadenopathy; 2. Generalized lymphadenopathy	0 or +1	0	1
Human herpesvirus-6 reactivation	Human herpesvirus-6 reactivation	0	0	0

**Figure 1:**
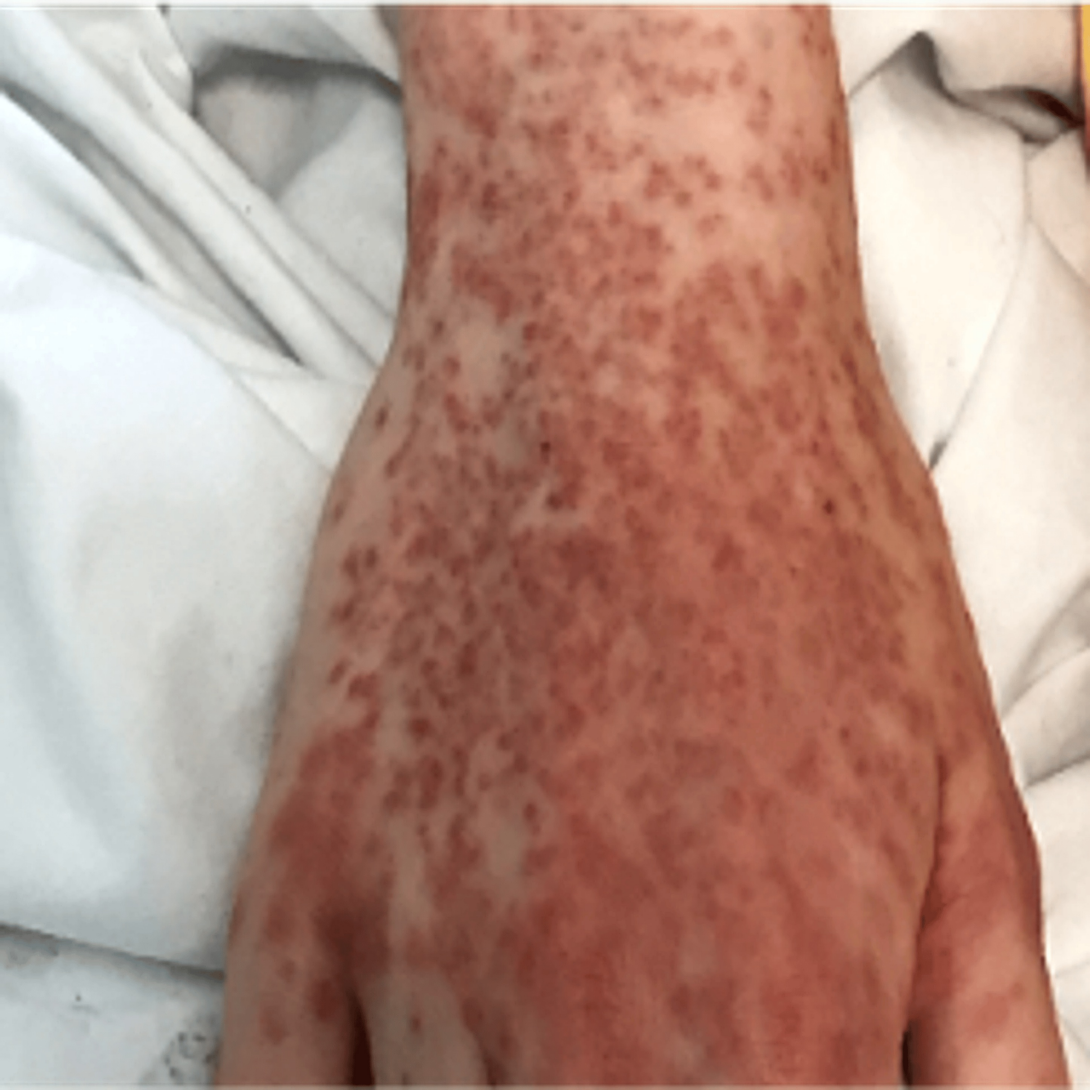
Maculopapular rash on our patient started on periphery.

**Figure 2:**
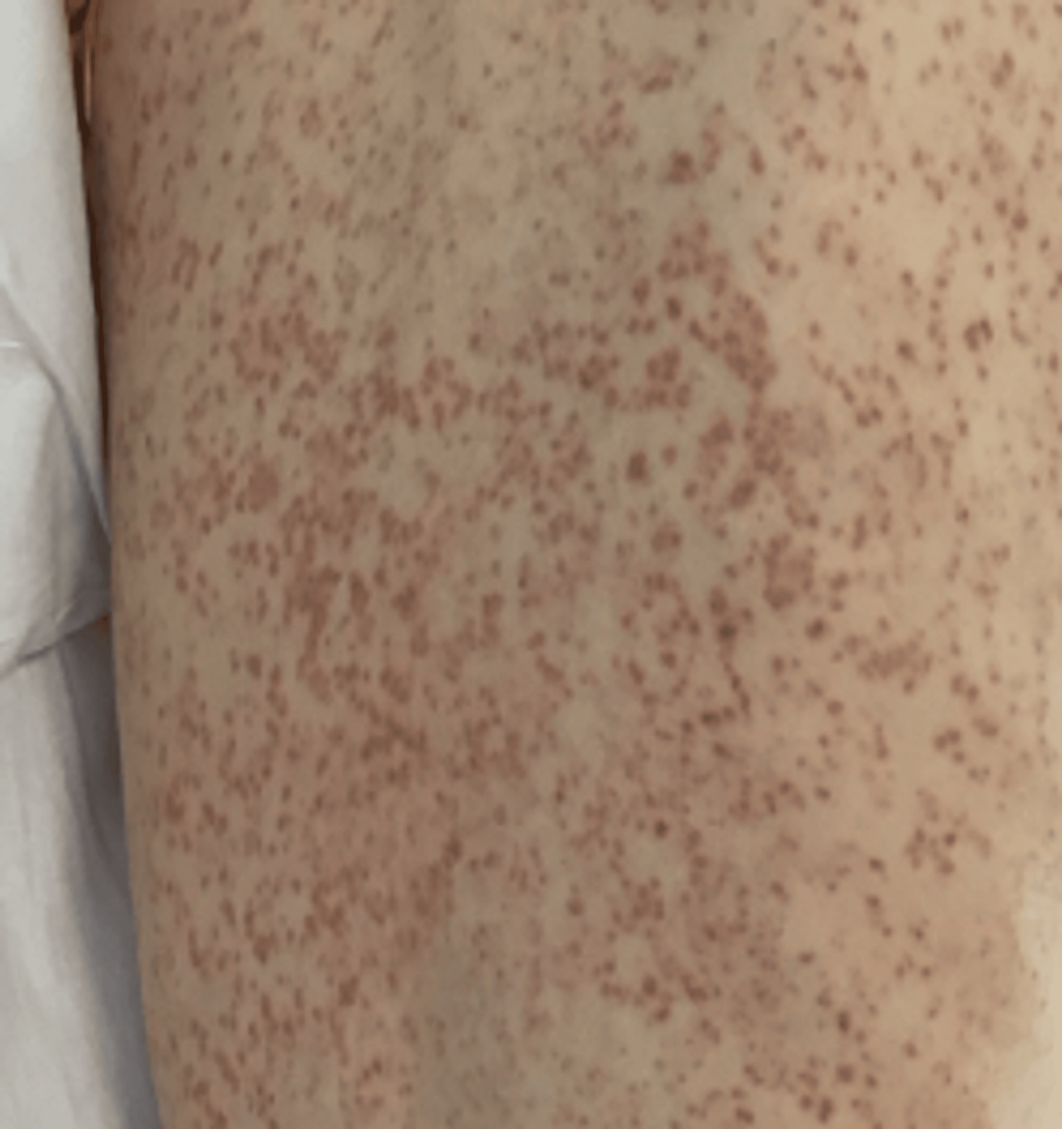
Maculopapular rash on our patient spreading to the trunk.

**Figure 3:**
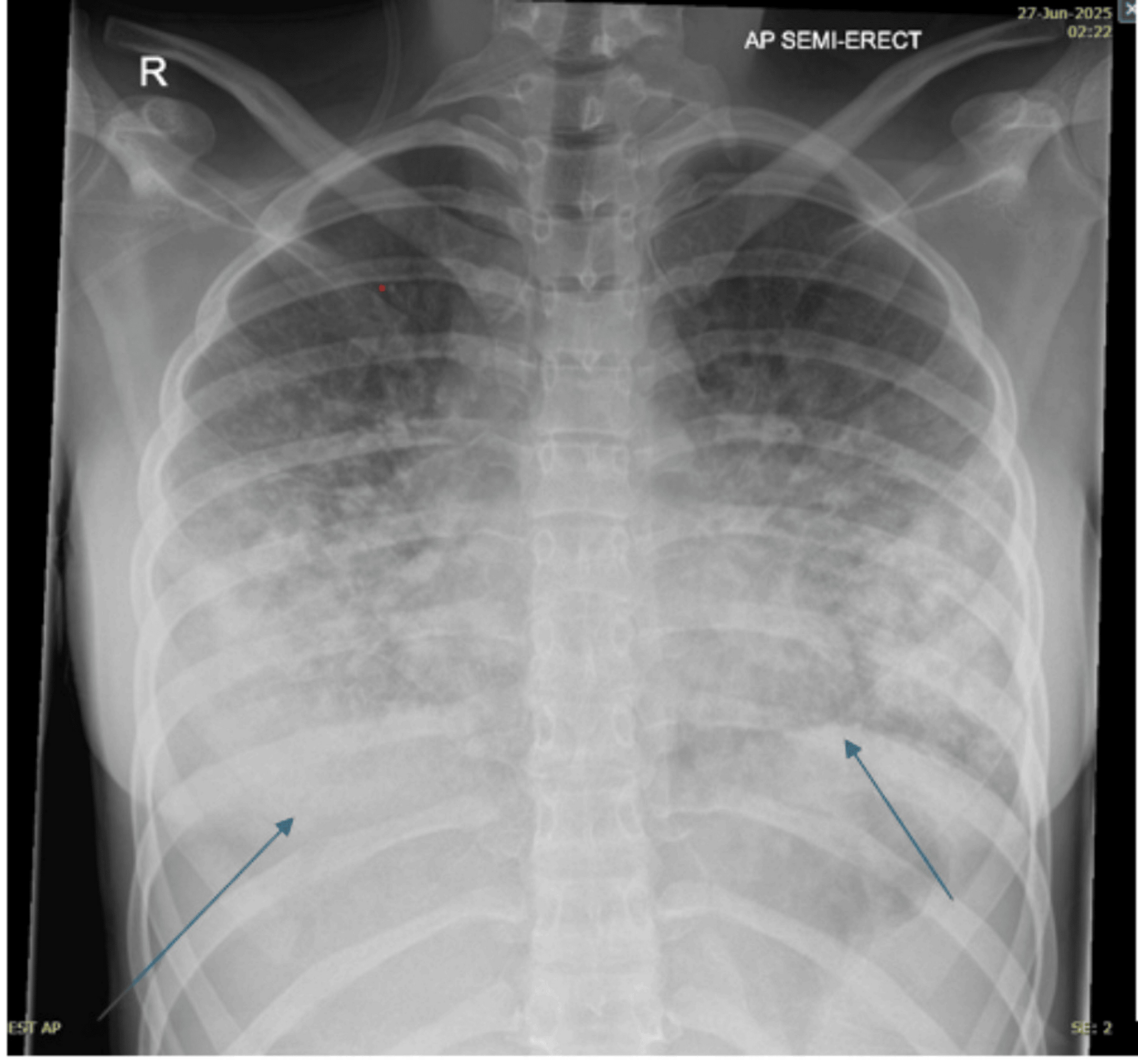
Chest X-ray of our patient showing inflammatory changes.

**Figure 4:**
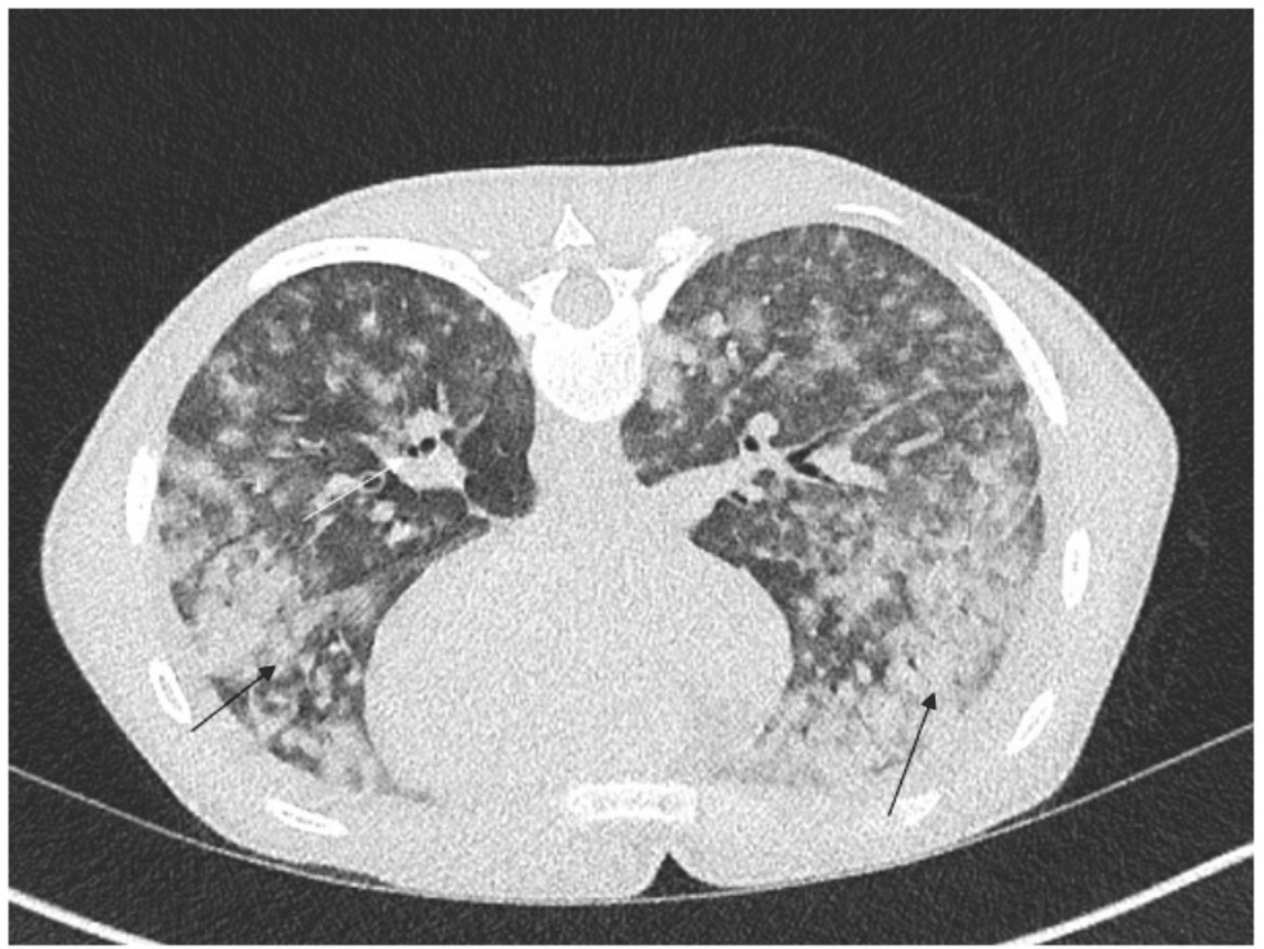
CT thorax of our patient showing diffuse consolidation with bronchiolar dilatation.

On examination, she was found to have a diffuse maculopapular rash (Figures 1, 2), which was evolving-every day, it was spreading and changing. It started as an eruption, but clinicians also described it as purpuric, followed by the formation of pustules. On respiratory examination, she had scattered bilateral chest crepitations. Cardiovascular and abdominal examinations were unremarkable. Laboratory investigations revealed raised inflammatory markers (see Table 2). Chest radiography (Figure 3) demonstrated changes consistent with pneumonia, while CT thorax (Figure 4) revealed bilateral infiltrates suggestive of either infection or drug-induced pneumonitis. 

**Table 2:** Laboratory findings of our patient throughout hospital stay.

	Units	Reference ranges	On discharge	Day 8	Day 6	Day 4	Day 3	Day 2	On admission
Hemoglobin	g/L	120-160	126	130	126	123	125	139	129
White blood cells	×10^9^/L	4.0-11.0	13.2	10.9	17.1	15	16.6	14.2	5.2
Neutrophils	×10^9^/L	2.0-7.7	10.97	8.62	8.61	9.26	8.49	8.90	3.07
Eosinophils	×10^9^/L	0.04-0.4	0.17	0.09	0.08	0.09	1.75	1.50	0.01
C-reactive protein	Mg/L	0-8	16	83	33	110	116	95	0.3
Albumin	g/L	36-48	35	35	23	24	23	23	35
Alkaline phosphatase	IU/L	30-125	74	110	209	231	227	157	59
Alanine aminotransferase	IU/L	5-45	71	256	235	288	237	113	14
Bilirubin	Umol/L	<21	13	11	07	06	09	09	22
Total protein	g/L	65-82	70	70	56	54	49	55	67
Urea	Mmol/L	3.0-7.6	5.5	3.7	3.0	2.5	3.1	2.9	4.2
Creatinine	Umol/L	55-87	54	53	55	75	69	76	69
Sodium	Mmol/L	135-144	138	140	138	136	135	135	139
Potassium	Mmol/L	3.5-5.3	5.3	3.5	4.2	4.5	4.4	4.3	4.0
Magnesium	Mmol/L	0.7-1.0	0.90		0.91		0.88		0.92

The differential diagnosis at this stage included community-acquired pneumonia versus drug-induced reaction to trimethoprim. She was started on intravenous antibiotics, trimethoprim was discontinued, and corticosteroid therapy was commenced. Despite this, her respiratory status deteriorated necessitating transfer to the intensive care unit for further management.

She was reviewed by the infectious disease team, and extended microbiological and serological investigations were negative (see Table 3). Based on clinical presentation, temporal association with trimethoprim, recurrence of symptoms upon steroid withdrawal, and absence of infectious etiology, a diagnosis of drug reaction with eosinophilia and systemic symptoms (DRESS) syndrome secondary to trimethoprim, complicated by pneumonitis, was established.

**Table 3:** Serological investigations of our patient. PCR: polymerase chain reaction.

Urine toxicology	Reference ranges	Negative
Herpes simplex virus PCR	Positive-negative	Negative
Varicella zoster virus PCR	Positive-negative	Negative
Adenovirus PCR	Positive-negative	Negative
Enterovirus PCR	Positive-negative	Negative
Human Herpesvirus 6 DNA screen	Positive-negative	Negative
Human Herpesvirus 7 DNA screen	Positive-negative	Negative
Hepatitis screen	Positive-negative	Negative

## Discussion

Drug reaction with eosinophilia and systemic symptoms (DRESS) is a rare but serious reaction to certain medications. It usually causes a widespread rash, fever, abnormal blood counts (often with high eosinophils), and inflammation of internal organs like the liver or kidneys. It happens in about one in 1,000-10,000 people taking high-risk drugs and can be fatal in up to 10% of cases [4,7]. While seizure medicines, allopurinol, and sulfa drugs are well-known causes, trimethoprim and its combination with sulfamethoxazole can also trigger DRESS but are less commonly recognized [8-10].

Our patient developed a maculopapular rash, fever, facial swelling, high white blood cell counts with eosinophilia, and elevated liver enzymes within a few days of starting trimethoprim. DRESS usually appears two to six weeks after starting a medicine, but in this case, it came sooner, which can happen if someone has been exposed to the drug before [10,11]. We used the RegiSCAR scoring system to confirm the diagnosis, which graded this case as (“probable”/“definite”). This scoring system is helpful to tell DRESS apart from other conditions that look similar, such as viral rashes, Stevens-Johnson syndrome, or sepsis [4,10].

DRESS is thought to happen when the body cannot fully break down the drug, leading to the buildup of toxic by-products that activate the immune system. Some cases are linked to reactivation of viruses like Human herpesvirus 6 (HHV-6) or Epstein-Barr virus (EBV) [4,11]. Sulfa drugs, including trimethoprim-sulfamethoxazole, are broken down in the liver into reactive compounds that may trigger a strong T-cell immune reaction in certain people [8,9].

Treatment focuses on stopping the drug right away. In more severe cases where organs are affected, systemic corticosteroids are often used, though randomized controlled evidence is limited and much guidance comes from case series and expert reviews [4,12]. Our patient improved with steroids/supportive care, and their rash and blood tests slowly returned to normal over six weeks. Steroids, if used, need to be tapered slowly to avoid relapse, which can happen in up to 10-15% of cases [7,12].

This case is another reminder that trimethoprim can cause DRESS. Doctors should keep this in mind when prescribing it, since early recognition and stopping the drug can prevent serious complications. More research is needed to find better treatments and understand why some people are more at risk than others [7-9].

## Conclusions

DRESS syndrome remains a diagnostic challenge due to its variable presentation and similarity to infectious and other inflammatory conditions. This case emphasizes that trimethoprim, though less commonly implicated than aromatic anticonvulsants or allopurinol, can induce DRESS with significant systemic involvement, including pneumonitis. Prompt recognition, immediate withdrawal of the culprit drug, and the timely initiation of corticosteroid therapy were crucial for a favorable outcome in our patient. Clinicians should be vigilant for early warning signs, particularly in patients who deteriorate following steroid withdrawal, as relapse is a known complication. Further research is warranted to optimize diagnostic criteria, clarify pathophysiology, and develop standardized treatment protocols for DRESS syndrome.
